# 2-NPPA Mitigates Osteoclastogenesis via Reducing TRAF6-Mediated c-fos Expression

**DOI:** 10.3389/fphar.2020.599081

**Published:** 2021-01-26

**Authors:** Zhihao Chen, Mina Ding, Eunjin Cho, Jihyoun Seong, Sunwoo Lee, Tae-Hoon Lee

**Affiliations:** ^1^ Department of Molecular Medicine, Chonnam National University Graduate School, Gwangju, South Korea; ^2^ Department of Oral Biochemistry, Dental Science Research Institute, School of Dentistry, Chonnam National University, Gwangju, South Korea; ^3^ Department of Chemistry, Chonnam National University, Gwangju, South Korea

**Keywords:** acetamide, osteoclastogenesis, bone loss, c-fos, TRAF6

## Abstract

Excessive bone resorption leads to bone destruction in pathological bone diseases. Osteoporosis, which occurs when osteoclast-mediated bone resorption exceeds osteoblast-mediated bone synthesis, is regarded a global health challenge. Therefore, it is of great importance to identify agents that can regulate the activity of osteoclasts and prevent bone diseases mediated mainly by bone loss. We screened compounds for this purpose and found that 2-(2-chlorophenoxy)-N-[2-(4-propionyl-1piperazinyl) phenyl] acetamide (2-NPPA) exhibited a strong inhibitory effect on osteoclastogenesis. 2-NPPA suppressed the mRNA and protein expression of several osteoclast-specific markers and blocked the formation of mature osteoclasts, reducing the F-actin ring formation and bone resorption activity. In a cell signaling point of view, 2-NPPA exhibited a significant inhibitory effect on the phosphorylation of nuclear factor kappa-B (NF-κB) and c-fos expression *in vitro* and prevented ovariectomy-induced bone loss *in vivo*. These findings highlighted the potential of 2-NPPA as a drug for the treatment of bone loss-mediated disorders.

## Introduction

Bone remodeling, which is a tightly regulated process, involves osteoclast-mediated bone resorption and osteoblast-induced bone formation (Feng and McDonald, 2011). In bone remodeling, old bone is resorbed and, simultaneously, the resorbed bone is replaced by newly synthesized bone; these processes are balanced by osteoclasts and osteoblasts (Clarke, 2008). Normally, to avoid excessive loss of bone mass, the dynamic of bone formation should be integrated closely with bone absorption to maintain a balance (Tanaka et al., 2005). Underlying pathological conditions such as excessive osteoclast-mediated bone resorption lead to excessive bone loss (Feng and McDonald, 2011). Osteoporosis, a common bone disease caused by excessive formation of osteoclasts and decreased bone mass, is caused by a reduced level of estrogen (Feng and McDonald, 2011). Bisphosphonate and its derivatives are the current and most popular clinically available drugs for the treatment of bone-loss related diseases. However, treatment with these drugs results in side effects such as jaw osteonecrosis and hypocalcemia (Drake et al., 2008; Do et al., 2012), suggesting a substantial need to identify new preventive drugs for the treatment of bone-loss related diseases.

Receptor activator of nuclear factor kappa-B (NF-κB) ligand (RANKL), a member of the tumor necrosis factor (TNF) superfamily, stimulates the development of osteoclast precursor cells into osteoclasts (Boyce and Xing, 2007). RANKL, which can be produced by marrow stromal cells and osteoblasts, has been suggested to correlate with the activation of osteoclast differentiation (Kearns et al., 2008). RANK, the receptor for RANKL, is a TNF receptor superfamily member located on the surface of osteoclast precursors and mature osteoclasts (Bezerra et al., 2005). In addition to RANKL, macrophage colony-stimulating factor (M-CSF) also has a critical function in osteoclast formation. M-CSF can induce osteoclast differentiation from its precursors and prolong the survival of mature osteoclasts (Wu et al., 2012).

C-Fos, a member of the Fos family, acts as an essential regulator in osteoclast differentiation (Boyce et al., 2005), and osteoclasts cannot form in the absence of c-fos (Wang et al., 1992). It regulates Fos genes expression and forms a complex that AP-1 with c-Jun, which also is required for the formation of osteoclast mediated by nuclear factor of activated T cells cytoplasmic 1 (NFATc1) (Boyce et al., 2005).

TRAF6 is a key mediator of the TNF receptor superfamily and interleukin-1/Toll-like receptor in proximal signaling (Lomaga et al., 1999); moreover, analysis of TRAF6-deficient mice has revealed the fundamental role of TRAF6 in osteoclast-mediated bone loss (Lomaga et al., 1999). In addition, recent biochemical evidence has indicated that after RANKL binds with RANK, TRAF6 is recruited, which is capable of activating RANKL-mediated signaling pathways through auto-activation, namely lys63-linked auto-ubiquitination, which subsequently activates the NF-κB signaling pathway (Adhikari et al., 2007; Lamothe et al., 2007a) and induces the expression of c-Src (Li et al., 2011; Wu et al., 2012). With the activation of TRAF6 and NF-κB, the main transcription factor of osteoclasts, i.e., NFATc1, is induced and triggers the expression of osteoclast-related genes such as cathepsin K (*CtsK*) and matrix metalloproteinase-9 (*MMP-9*) (Delaisse et al., 2003; Kim and Kim, 2014; Amarasekara et al., 2018). This confirmed the important roles of the activation of TRAF6 and NF-κB in the formation of mature osteoclasts. Currently, the popular used clinical drugs for treating bone loss-related diseases are bisphosphonate and its derivatives. (Papapetrou, 2009; Solomon et al., 2009) However, side effects such as jaw and hypocalcemia osteonecrosis always are associated with their treatment (Papapetrou, 2009; Solomon et al., 2009), which emphasizes the need for the discovery of new drugs to anti-bone loss mediated disease.

To identify candidates for anti-osteoclastogenesis, an initial screen was performed of our in-house-synthesized compounds (Chen et al., 2019b). After comparison with compounds with previously established *in vitro* and *in vivo* anti-osteoclastogenesis activity assay, 2-NPPA was identified as an effective inhibitor of osteoclastogenesis. *In vitro*, 2-NPPA reduced the protein level of several osteoclast-specific markers and the phosphorylation of NF-κB. Overexpression of c-fos, to some degree, diluted the inhibitory effect of osteoclast differentiation via 2-NPPA treatment. In addition, *in vivo* experiments suggested that treatment of 2-NPPA significantly prevented ovariectomy (OVX)-induced bone loss. Collectively, these results suggested that 2-NPPA suppressed the formation of mature osteoclasts *in vitro* and inhibited bone loss *in vivo*, highlighting its prospective use for the treatment of bone loss-related diseases caused by alterations in the formation of mature osteoclasts.

## Materials and Methods

### Materials and Reagents

2-NPPA (purity > 99%, ChemBridge ID#7628815), PPOA-*N*-Ac-2-Cl (purity > 99%, ChemBridge ID#9026405), PPOAC-Me (purity > 99%, ChemBridge ID# 7932051), PPOAC-Ac (purity > 99%, ChemBridge ID# 9021720) and PPOAC-Bz (purity > 99%, ChemBridge ID#7933842) were obtained from ChemBridge, 11,199 Sorrento Valley Rd, suite 206 San Diego, CA, United States, dissolved in dimethyl sulfoxide (Sigma-Aldrich, St. Louis, MO, United States), and stored at -20 °C. MG132 got from Sigma-Aldrich, St. Louis, MO, United States. The following primary antibodies were obtained: anti-β-actin (#A5441) from Sigma-Aldrich, St Louis, MO, United States; anti-cathepsin K (#48353), anti-TRAF6 (B-10), and anti-c-Src (#sc-8056) from Santa Cruz Biotechnology, Inc., Dallas, TX, United States; anti-ubiquitin, lys63-specific (#05-1308) from EMD Millipore Corp; and anti-c-fos (#4384s), anti-phospho-NF-κB (#s536), anti-NF-κB (#4764s), anti-ubiquitin (#3936s), anti-NFATc1 (#8032s), anti-phospho-p38 (#4511s), anti-phospho-ERK1/2 (#4370s), anti-phospho-JNK (#9255s), anti-phospho-AKT (#4060s), anti-phospho-IκBa (#2859s), anti-p38 (#9212s), anti-ERK1/2 (#9102s), anti-JNK (#9252s), anti-AKT (#9272s), and anti-IκBa (#9242s) from by Cell Signaling Technology, Boston, MA, United States. Horseradish peroxidase (HRP)-conjugated secondary antibody (#7074S) was supplied by Cell Signaling Technology, Boston, MA, United States. The ECL system (#RPN2106) used for the detection of chemiluminescence signals was obtained from iNtRON, Seoul, Korea. The BCA protein assay kit was purchased from Pierce Biotechnology, Rockford, IL, United States. The TRAP staining assay and bone resorption assay kit were obtained from Cosmo Bio Co., Ltd., Tokyo, Japan. RANKL and M-CSF were procured from Peprotech, Rocky Hill, NJ 08553 United States. Alpha-modified minimal essential medium and fetal bovine serum were purchased from Thermo Fisher Scientific, Waltham, MA, United States.

### Bone Marrow-Derived Macrophage Isolation and Culture

For the *in vitro* osteoclastogenesis assay, mice bone marrow-derived macrophages (BMMs) were collected as previously described (Chen et al., 2019b). Briefly, after euthanasia 8-week-old C57BL/6J mice in CO_2_ condition box, BMMs were isolated from the mouse tibias and femurs by flushing the bone marrow with α-MEM. The flushed cells were collected and cultured in α-MEM supplemented with 10% heat-inactivated FBS and 1% penicillin/streptomycin. During the morning of the second day, the non-adherent cells were collected and cultured in Petri dishes with M-CSF (30 ng/ml) to select BMMs. After incubation for 3 days, the adherent cells (BMMs) were detached using a cell-free enzyme and collected for further culture in an induction medium to induce osteoclast differentiation. All animal experiments were approved by IACUC at Chonnam National University (approval number: CNU IACUC-YB-2019-46).

### *In vitro* Osteoclastogenesis and Cell Viability Assay

BMMs (2 × 10^4^ cells per well) were cultured in 48-well plates and treated with 30 ng/mL M-CSF and 50 ng/ml RANKL until mature osteoclasts were observed in the DMSO treatment group. Next, the osteoclasts were fixed in 4.0% formaldehyde for at least 15 min and stained using the TRAP-staining kit. The spread of the osteoclast area and the number of osteoclasts formed were counted using ImageJ software (NIH, Bethesda, MD), based on the number of nuclei per cell (*n* ≥ 3) visible under a microscope (Ahn et al., 2018).

The viability of BMMs was assessed after incubation with 2-NPPA using a cell viability assay kit. BMMs (1 × 10^4^ cells/well) were cultured overnight in a 96-well plate. After RANKL, M-CSF, and various concentrations of 2-NPPA were supplemented to the medium, the cells were incubated at 37 °C in 5% CO_2_ for 72 h; the medium was replaced with FBS-free medium containing 10% cell viability reagent. The cells were incubated at 37 °C in 5% CO_2_ for 30 min, and the absorbance at 450 nm of each well was measured using a SpectraMax i3x microplate reader (Molecular Devices, San Jose, CA, United States) (Chen et al., 2019b).

### Plasmid Transformation for *c-Fos* Gene Expression

For overexpression of c-fos, *pIRES-hrGFP-2a* (empty vector) or *pIRES-hrGFP-c-fos-2a* (c-Fos vector) plasmid were transformed into RAW264.7 cells using Lipofectamine 2000 Reagent (Thermo Fisher Scientific, Waltham, MD, United States) in a culture medium for 24 h; they were then seeded in 6-well plate and/or 48-well plate with the treatment of RANKL (50 ng/ml) and/or different indicated dose of 2-NPPA.

### Bone Resorption and F-Actin Ring Immunofluorescence Assay

A bone resorption assay kit was used to evaluate the osteoclast bone resorption activity in the light of the manufacturer’s directions. BMMs (2 × 10^4^ cells/well) were cultured in the kit-supplied coated-plate supplemented with M-CSF. On the next day, the medium was replaced, and the cells were incubated with M-CSF and RANKL and treated with or without the indicated concentrations of 2-NPPA until the formation of mature osteoclasts was observed. On the next day, the supernatant in each well was harvested, placed into a black polypropylene 96-well microplate, and mixed with NaOH (#S5881). The fluorescence intensity of each well was measured using a SpectraMax i3x fluorescence plate reader (excitation wavelength: 485 nm; emission wavelength: 535 nm) (Chen et al., 2019b). The demineralized pit areas were calculated from 10 randomly selected images per well using ImageJ software, as previously described (Chen et al., 2019b).

The F-actin rings were detected using a rhodamine-conjugated phalloidin staining assay (Thermo Fisher Scientific). BMMs were seeded on 12-mm cover glasses in the presence or absence of 2-NPPA. After mature osteoclasts were observed, the cells were fixed for at least 15 min in 4.0% paraformaldehyde and incubated in 5% FBS for 60 min at room temperature to block non-specific binding. After the cells were washed in cold PBS, rhodamine-conjugated phalloidin (1:40) was added to each well to visualize the F-actin rings. After 20 min, DAPI solution (1:5,000) was added to each well and incubated for 5 min, and the cells were washed three times with PBS and observed using a fluorescence microscope (Ahn et al., 2018).

### RNA Isolation and Quantitative Real-Time PCR

Total RNA from the BMMs was isolated using a QIAzol RNA lysis reagent (Qiagen Sciences, Valencia, CA, United States) after culture in a 6-well plate with or without 2-NPPA at the indicated concentrations for 3 days in the induction medium, as used in our previous study (Chen et al., 2019b). PrimeScript™ RT reagent kit for qRT-PCR (Takara Biotechnology, Tokyo, Japan) was used to synthesize cDNA in accordance with the manufacturer’s protocol, and real-time PCR was performed using a QuantStudio 3 qRT-PCR system (Applied Biosystems, Foster City, CA, United States) with Power SYBR Green PCR Master Mix (Applied Biosystems, Foster City, CA, United States) and a temperature protocol provided by the manufacturer (Chen et al., 2019b). The cycle threshold values obtained were expressed as relative ratios and calculated using the 2^−ΔΔ^ CT method; as per the method in our previous study, mRNA expression was normalized to the expression of glyceraldehyde 3-phosphate dehydrogenase (GAPDH) (Chen et al., 2019b). The primers used for real-time PCR assay are listed in Supplementary Table S1.

### Western Blotting Assays

Osteoclasts were lyzed in RIPA buffer (#89900; Thermo Fisher Scientific). After lysis, the cells were centrifuged at 16,400 g at 4 °C for 30 min, and the pelleted material was discarded. After measurement of the protein concentration of the supernatant following whole cell lysis using the BCA protein assay, the boiled samples were loaded onto a 12% SDS-page gel and electrophoresed. The separated proteins were transferred to a PVDF membrane. Non-specific binding to the membrane was blocked by incubation of the membrane in 5% skim milk for 1.5 h, and the membranes were incubated with the appropriate primary antibody (1:1000 dilution) at 4 °C overnight. After the PVDF membrane was washed three times with TBST, the membrane was incubated for 1 h at room temperature with HRP-conjugated secondary antibody (1:2000 dilution), and ECL reagent was applied to detect the chemiluminescence signals in accordance with the manufacturer’s protocol (Chen et al., 2019b).

### OVX-Induced Osteoporosis Mice Model

All mice were grown in a specific-pathogen-free facility. To evaluate the effect of 2-NPPA on osteoclastogenesis *in vivo*, we established an OVX-induced bone loss model. Briefly, 30 healthy 7-week-old C57BL/6 female mice were allocated to one of three groups (sham, control, and treatment). The mouse in the control and treatment groups received OVX surgery, and the sham group mice received only an abdominal incision. After recovery for 1 week, mice in the treatment group received intraperitoneal injection of 2-NPPA (10 mg/kg, dissolved in a mixture of 5% Tween 80 and 5% DMSO) every other day for 5 weeks. The sham and control groups received an equal volume of a mixture of 5% Tween 80 and 5% DMSO. The mice were weighed every week. On the final experimental day, after the mice were euthanized, the serum was collected for biochemical analysis (CTX-1), and both the femur and tibia were fixed in 4.0% paraformaldehyde after removal of the surrounding tissues for further analysis by microcomputed tomography (micro-CT).

### Microcomputed Tomography (Micro-CT) Scanning and Histological Analysis

A Quantum GX Micro-CT imaging system (PerkinElmer, Hopkinton, MA, United States) located at Korea Basic Science Institute (Gwangju, Korea) was used for performing the CT imaging. The fixed tibias and femurs were analyzed using a high-resolution micro-CT instrument; the BV, BV/TV, Tb.Th, Tb.V, BMD, and Ct.V parameters were measured. Following micro-CT analysis, the femurs were decalcified in 20% EDTA (Sigma-Aldrich) at 4 °C for 5 days and then embedded in paraffin to prepare sections for further histological analysis. Subsequently, the samples were deparaffinized, and TRAP staining was performed.

### Statistical Analysis

Unpaired two-tailed Student’s *t*-test (*p < 0.05; **p < 0.01; ***p < 0.001; NS, not significant) was performed for statistical analyses. All data are expressed as the mean ± standard deviation (SD). The results are representative examples of at least three independent experiments.

## Results

### 2-NPPA Attenuated RANKL-Induced Osteoclastogenesis With Little Effect on the Osteoblast Differentiation *in vitro*

**FIGURE 1:**
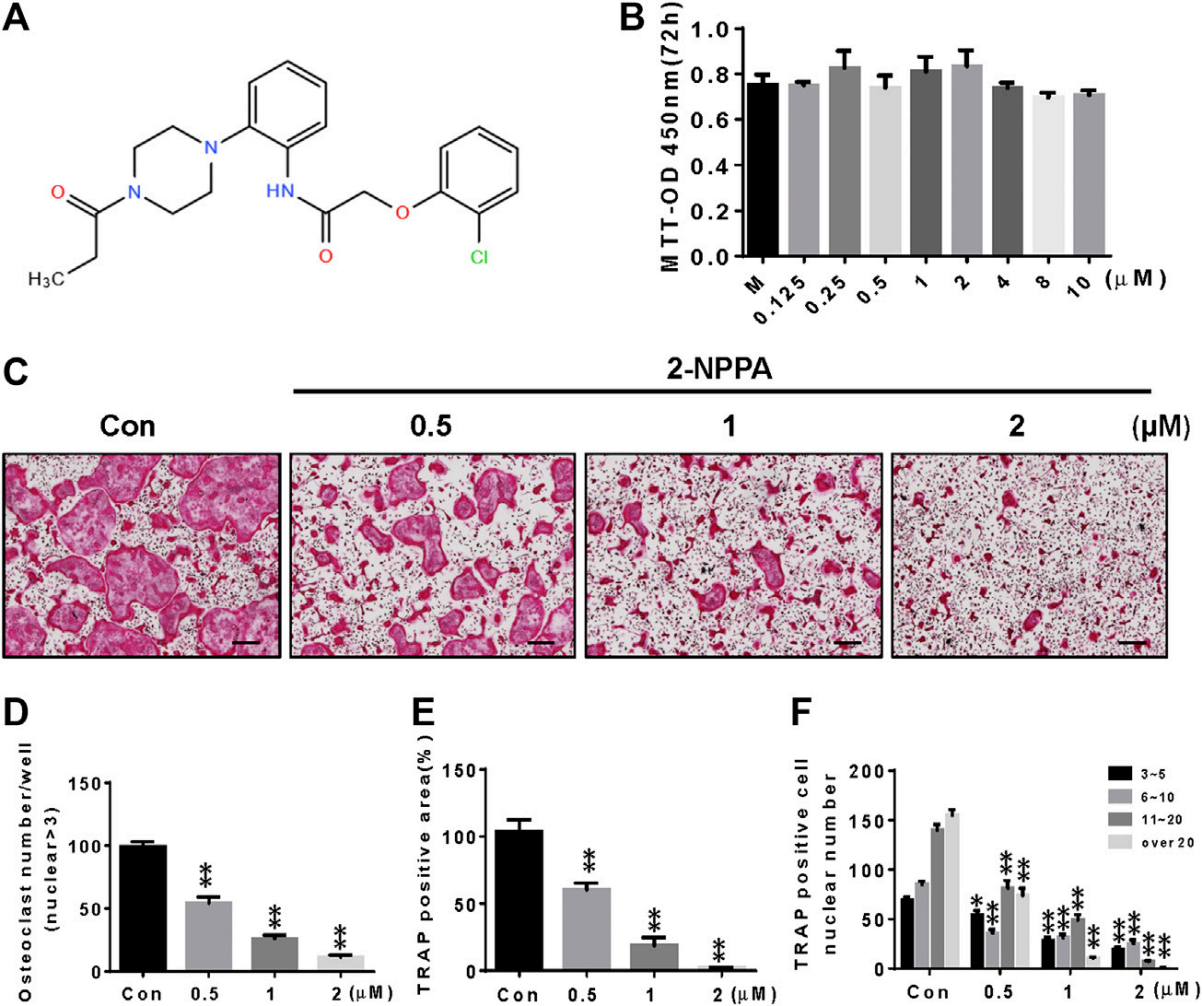
2-NPPA attenuated RANKL-induced osteoclastogenesis *in vitro*. **(A)** The structure of 2-NPPA. **(B)** The MTT assay was performed after treatment for 72 h. **(C)** The inhibitory effect of 2-NPPA on osteoclast differentiation was examined using TRAP staining. BMMs were treated with various doses of 2-NPPA (0, 0.5, 1, and 2 μM) for 3 days in the induction medium with 30 ng/mL M-CSF and 50 ng/ml RANKL; subsequently, the TRAP staining assay was performed to examine osteoclast formation. The number of osteoclasts per well **(D)**, area **(E)**, and the number of cells per well of TRAP-positive multinuclear cells **(F)** were calculated. All experiments were repeated three times. Bars represent means ± SD. *p < 0.05, **p < 0.01, and ***p < 0.001 vs. vehicle-treated control (Con; 0 μM 2-NPPA); “M” indicates M-CSF; “Con” indicates M-CSF and RANKL treatment. Scale bar = 200 μm.

After comparison with compounds with previously established *in vitro* and *in vivo* anti-osteoclastogenesis activity (Supplementary Figure S1), 2-NPPA (Figure 1A) was further identified as the strongest inhibitor of osteoclastogenesis. The area, number of osteoclast cells, and number of osteoclast cells of different nuclei per cell decreased more dramatically in the 2-NPPA treatment groups than in the Con (control) group (Figures 1C–F), suggesting that 2-NPPA can serve as a candidate inhibitor of osteoclastogenesis without cytotoxic effects (Figure 1B). In addition, to examine whether 2-NPPA influenced osteoblastogenesis *in vitro*, BMP-induced differentiated osteoblasts were treated with different dose of 2-NPPA and examined using AR staining (Supplementary Figures S2A,B) and real-time PCR assay (Supplementary Figures S2C,D). Results suggested that 2-NPPA have a little effect on BMP2-induced osteoblast differentiation *in vitro* (Supplementary Figure S2).

### 2-NPPA Inhibited RANKL-Induced F-Actin Ring Formation in a Dose-dependent Manner and Affected the Early Stage of Osteoclast Differentiation

**FIGURE 2:**
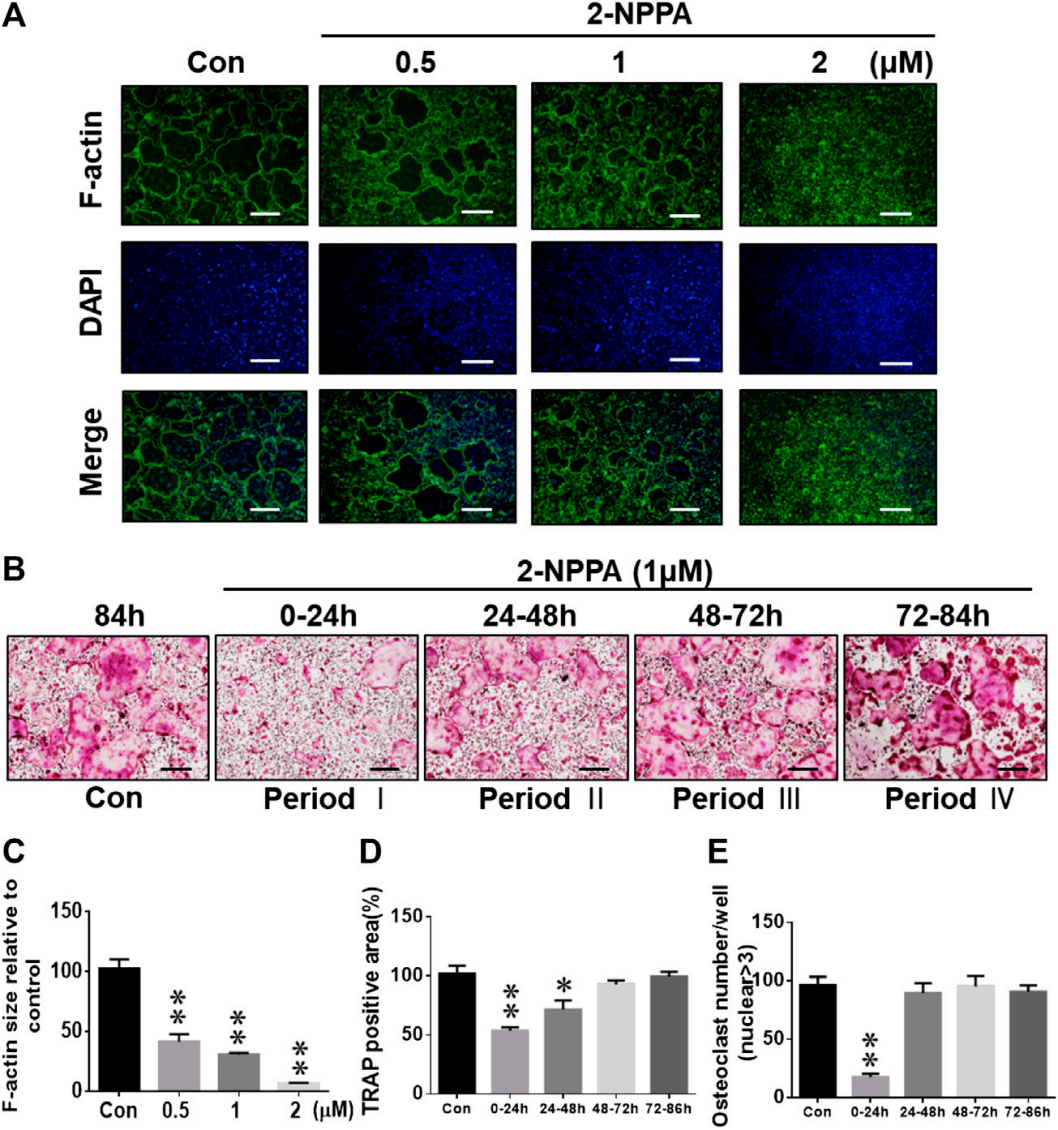
2-NPPA inhibited the RANKL-induced formation of F-actin rings and altered osteoclast differentiation at the early stages. **(A)** Visualization of actin ring formation during osteoclastogenesis in the presence of the indicated concentrations of 2-NPPA or Con. **(C)** The calculation of F-actin ring size in panel A performed using ImageJ. **(B)** BMMs were divided into five groups (Con, and Periods I, II, III, and IV) and grown in culture medium containing 30 ng/mL M-CSF and 50 ng/ml RANKL for 4 days. The BMMs in Periods I–IV groups were exposed to 2-NPPA for 24 h on different days. After 4 days, the cells in each group were fixed and TRAP staining was performed to determine the presence of osteoclasts. The calculations of the area **(D)** and the total number of osteoclasts **(E)** in panel B were performed using ImageJ software. All experiments were repeated three times. Bars represent means ± SD. *p < 0.05, **p < 0.01, and ***p < 0.001 vs. vehicle-treated control (Con, 0 μM 1-NPPA); “Con” indicates M-CSF and RANKL treatment. Scale bar = 200 μm.

We performed an immunofluorescence assay to explore the effect of 2-NPPA on the formation of actin rings in RANKL-induced osteoclastogenesis *in vitro*. Actin rings were well-formed after stimulation with M-CSF and RANKL in the control group, whereas there was a significant decrease in the size and formation of F-actin rings after treatment with the indicated dose of 2-NPPA (Figure 2A). To further understand the inhibitory role of 2-NPPA in osteoclastogenesis, 1 µM 2-NPPA was supplied to the osteoclast induction medium at various time points during the process of osteoclast formation. As shown in Figures 2B–E, osteoclast formation was dramatically suppressed after treatment with 2-NPPA in the early stages of osteoclast formation (0–24 h); however, no significant difference was found after 2-NPPA treatment in the late stages, 48–72 h and 72–84 h (Figure 2B), suggesting that 2-NPPA inhibited osteoclast formation in the early stages of osteoclastogenesis, subsequently reducing the formation of F-actin rings.

### 2-NPPA Suppressed the Function of Osteoclasts and Osteoclast-specific Gene Expression

**FIGURE 3:**
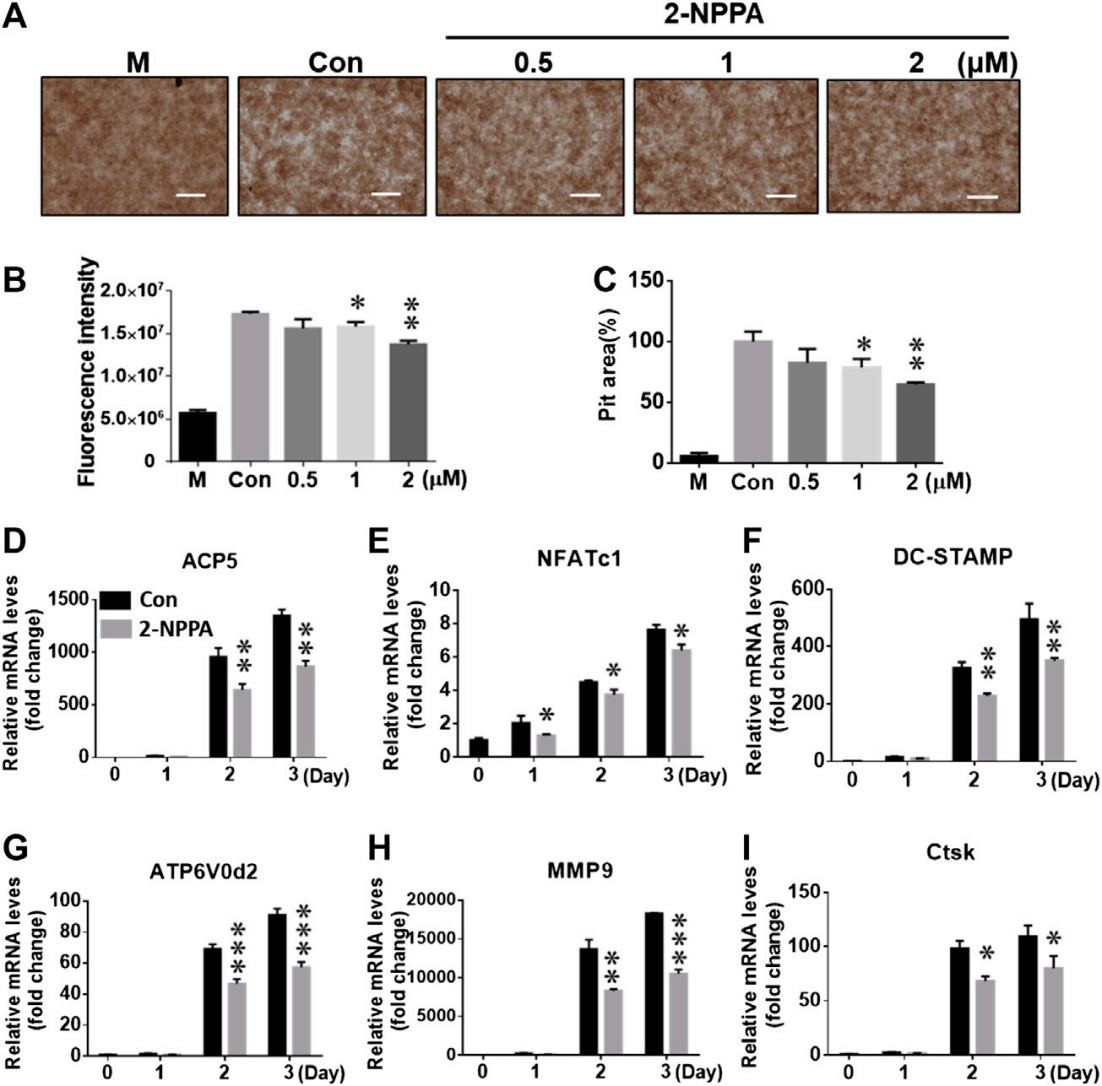
2-NPPA suppressed the function of osteoclasts and osteoclast-specific gene expression. **(A–C)** BMMs were cultured in a fluoresceinamine-labeled calcium phosphate plate and treated with the indicated concentrations of 2-NPPA for 6 days. After 6 days, the fluorescence intensities in all groups were measured at excitation and emission wavelengths of 485 and 535 nm, respectively, using a fluorometric plate reader **(B)**. The demineralization in the calcium phosphate was measured and the demineralized pit area was calculated using ImageJ **(C)**; scale bar = 200 μm. **(D–I)** 2-NPPA inhibited the expression of osteoclast-specific markers induced via RANKL. The relative mRNA expression of the indicated osteoclast genes were analyzed using qRT-PCR in the presence/absence of 1 µM 2-NPPA for 0, 1, 2, or 3 days. Black bars indicate the control (“Con”), and gray bars indicate the 2-NPPA-treated group. Transcript levels were normalized to the expression of the control group at Day 0. The qRT-PCR primers using in this study are listed in Supplementary Table S1. All experiments were repeated three times. Bars represent means ± SD. *p < 0.05, **p < 0.01, and ***p < 0.001 vs. the vehicle-treated control (Con, 0 μM); “M” indicates M-CSF treatment.

A bone resorption assay was used to confirm the *in vitro* effect of 2-NPPA on osteoclast-mediated bone resorption, and the pit areas were imaged using a light microscope (Figure 3A). The results suggested that the size of the bone resorptive area was enhanced more markedly (Figures 3A,C) after treatment with M-CSF and RANKL than after treatment with the negative control (“M,” only treated with M-CSF). In addition, the percentage of the bone resorption area was decreased significantly after treatment with 1 and 2 µM of 2-NPPA. As shown in Figure 3B, the resorption-related fluorescence intensity also decreased along with an increase in the treatment dose. Together with the effect of 2-NPPA on the formation of mature osteoclasts, these results indicated that the 2-NPPA-mediated inhibition of bone resorption was attributable to the impaired formation of mature osteoclasts.

To explore the role of 2-NPPA in the process of osteoclastogenesis, real-time PCR assay was used to analyze gene expression. As shown in Figures 3D–I, mRNA expression level of the indicated osteoclast-specific marker genes increased markedly upon treatment with M-CSF and RANKL, as shown in the control group (black bars). 2-NPPA significantly reduced the RANKL-mediated transcription of the indicated genes (gray bars), which further supported the inhibitory effect of 2-NPPA on the formation and function of osteoclasts.

### 2-NPPA Suppressed Osteoclast-specific Protein Expression and the Phosphorylation of NF-κB/I-κBα, Without Interfering MAPK and AKT Signaling Pathways

**FIGURE 4:**
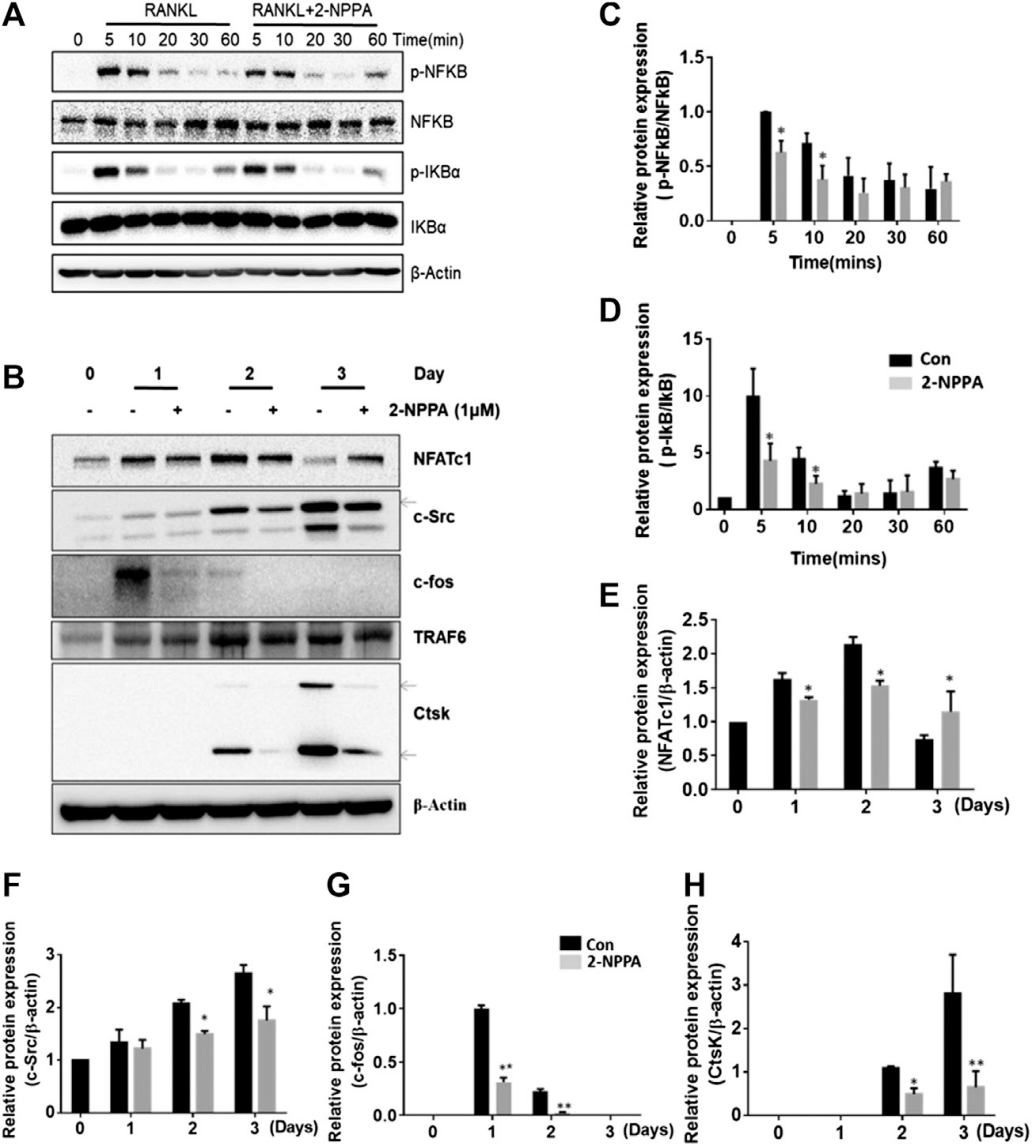
2-NPPA suppressed RANKL-induced c-fos expression. **(A)** BMMs were treated with 100 ng/ml RANKL for 0, 5, 10, 20, 30, or 60 min together with 1 μM 2-NPPA or DMSO, and the phosphorylation of NF-κB and IκBa were analyzed by using immunoblotting. **(B)** BMMs were cultured with 1 μM 2-NPPA for 0, 1, 2, or 3 days in the inductive medium. After the cell lysates were processed, immunoblotting was performed with the indicated antibodies. β-actin was used as the loading control. **(C–H)** The densitometry graphs of **(A,B)**; β-actin was used as a loading control. *p < 0.05, **p < 0.05 vs. the vehicle-treated control, 0 μM. All experiments were repeated three times. Bars represent means ± SD.

Next, we aimed to understand the mechanism underlying the inhibitory effects of 2-NPPA on the formation and functions of osteoclast. Results, shown in Figure 4B, suggested that after 2-NPPA treatment, the expressions of the osteoclast-specific protein induced by RANKL were significant suppressed, especially, the protein expression of c-fos. Interestingly, the expression of NFATc1, a key transcription factor in osteoclastogenesis, was significant reduced in the 2-NPPA treatment groups than in the positive control group (without 2-NPPA treatment) during Day1 and Day2. However, a higher level of NFATc1 was appeared at Day3. In addition, the protein level of Ctsk, a main resorption protease in osteoclastogenesis (Troen, 2004), was also dramatically reduced after treatment with 2-NPPA (Figure 4B). Activation of the NF-κB, MAPK and AKT pathways were regarded as the main signaling pathways during osteoclastogenesis (Walsh et al., 2008; Tang et al., 2009; Lee et al., 2016; Chen et al., 2019a), and they mediated the expression of NFATc1 (Takayanagi et al., 2002; Boyce et al., 2015). To investigate the mechanisms underlying the effect of 2-NPPA on the inhibitory effect of osteoclast differentiation, we examined the phosphorylation of NF-κB, I-κBα, JNK, ERK, p38, AKT and the degradation of I-κBα within 1 h after RANKL stimulation. A time-course analysis suggested that RANKL led to a large increase in the protein expression levels of phosphorylated NF-κB and I-κBα, which were inhibited by 2-NPPA treatment (Figure 4A), leaving p-JNK, p-ERK, p-p38, p-AKT and the degradation of I-κBα unaffected. Collectively, these results indicated that 2-NPPA suppressed RANKL-induced osteoclastogenesis by reducing the phosphorylation of NF-κB, I-κBα and the c-fos expression which subsequently resulted in the delayed protein expression of NFATc1 and downregulation of the protein expression of Ctsk.

### 2-NPPA Showed Slightly Effect on the TRAF6 or TRAF6-Mediated lys63 Linked-Ubiquitination

Our results collected so far suggested that 2-NPPA suppressed osteoclast differentiation via inactivation of the NF-κB pathway. However, the mechanism of the pleiotropic actions of 2-NPPA on the activation of NF-κB was unclear. TRAF6, a widely accepted upstream regulator of NF-κB, mediated the activation of NF-κB pathway in response to RANKL stimuli (Lomaga et al., 1999; Wu and Arron, 2003; Lamothe et al., 2007a; Lamothe et al., 2007b). In addition, the activation of TRAF6 is mediated by its ubiquitination (Lamothe et al., 2007a; Lamothe et al., 2007b). As 2-NPPA had no effect on the mRNA and protein expression of TRAF6 (Figure 4B, Supplementary Figure S5A), we hypothesized that the inhibitory effect of 2-NPPA on osteoclast differentiation may occur through inactivation of TRAF6, via a reduction in its or it mediated ubiquitination. Indeed, the activation of TRAF6 is specifically mediated by lys63-linked auto-ubiquitination, a well-known mechanism through which activation of the downstream signaling occurs (Lamothe et al., 2007a; Lamothe et al., 2007b). Therefore, we examined the total ubiquitination of TRAF6 and the lys63-specific ubiquitinated-TRAF6 in the presence of RANKL with or without treatment with 2-NPPA (1 μM). Remarkably, RANKL led to a significant increase in the ubiquitination of TRAF6, especially the lys63-specific ubiquitinated-TRAF6, whereas there was a slightly decrease in the 2-NPPA treatment group (Supplementary Figures S5B–D). Overall, our cellular experiments indicated that 2-NPPA was predicted to repress osteoclastogenesis partly via native regulation of the activation of the critical adaptor protein TRAF6, by reduce its or it-mediated Lys 63-linked ubiquitination.

### Overexpression of c-Fos Diluted the Inhibitory Effect of Osteoclast Differentiation by 2-NPPA Treatment

**FIGURE 5:**
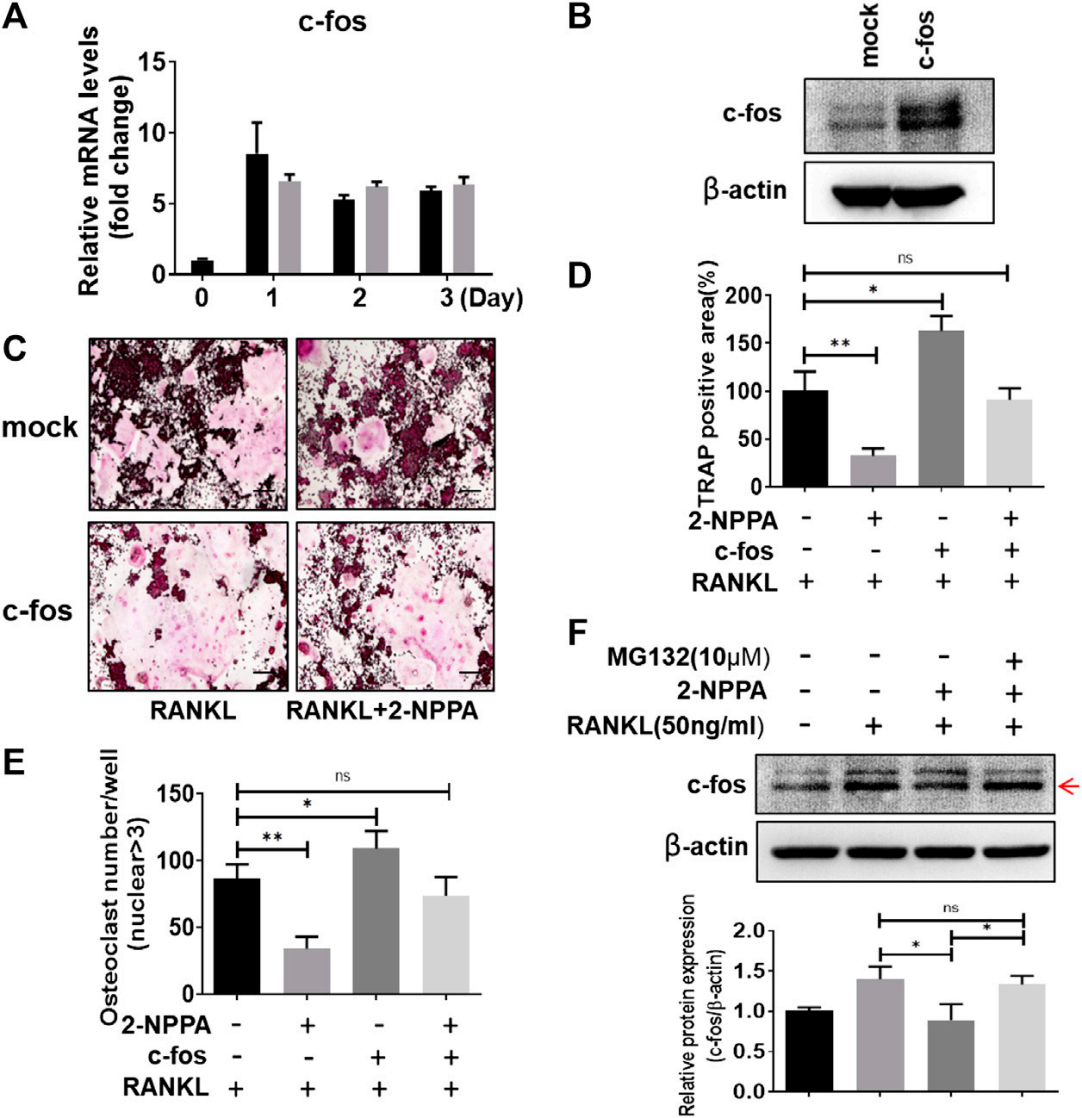
Overexpression of c-fos diluted the inhibitory effect of osteoclast differentiation by 2-NPPA treatment. **(A)** Real-time PCR was performed to examine the mRNA level of the c-fos in BMMs. **(B)** RAW264.7 cells were transfected with empty vector (mock) or c-fos vector (c-fos), the expression of c-fos was analyzed by using Western Blotting. β-actin was used as the loading control. **(C–E)** The transient transformed RAW264.7 cells (*mock* and *c-fos*) were seeded and then incubated with or without 2-NPPA (1 μM) in the presence of RANKL (50 ng/ml). After 4 days, the cells was fixed and stained for the TRAP assay **(C)**. **(D–E)** The TRAP-positive area and multinucleated osteoclast number were counted. **(F)** c-fos expression in the indicated groups was analyzed and the relative protein expression level was quantitated using ImageJ. *p < 0.05, **p < 0.01; scale bar = 200 μm. All experiments were repeated three times. Bars represent means ± SD.

C-fos acted as an essential regulator in the formation of osteoclast, and osteoclasts cannot form without c-fos (Wang et al., 1992). Therefore, RAW264.7 cells were transformed with mock vector or c-fos expression vector (Figure 5B) for examine if overexpression of c-fos diluted the osteoclast differentiation in the treatment of 2-NPPA. Our data in Figures 4B,5A showed that the protein expression of c-fos was greatly reduced after 2-NPPA treatment without interference of its mRNA expression, which guided us to hypothesize that the inhibitory effect of 2-NPPA on mature osteoclast formation is due to the defective of c-fos. TRAP staining assay indicated that 2-NPPA (1 μM) treatment significantly reduced the formation of osteoclast in mock vector transformed cells. However, the overexpressed c-fos partly abolished the inhibitory effect of 2-NPPA on osteoclast formation (Figures 5C–E). In addition, MG132, a proteasome inhibitor, abolished the effect of c-fos depletion by 2-NPPA treatment (Figure 5F). These results indicated that 2-NPPA suppressed osteoclast differentiation by reduction of the protein level of c-fos through proteasomal degradation.

### Attenuation of OVX-Induced Bone Loss via 2-NPPA Treatment *in vivo*

**FIGURE 6:**
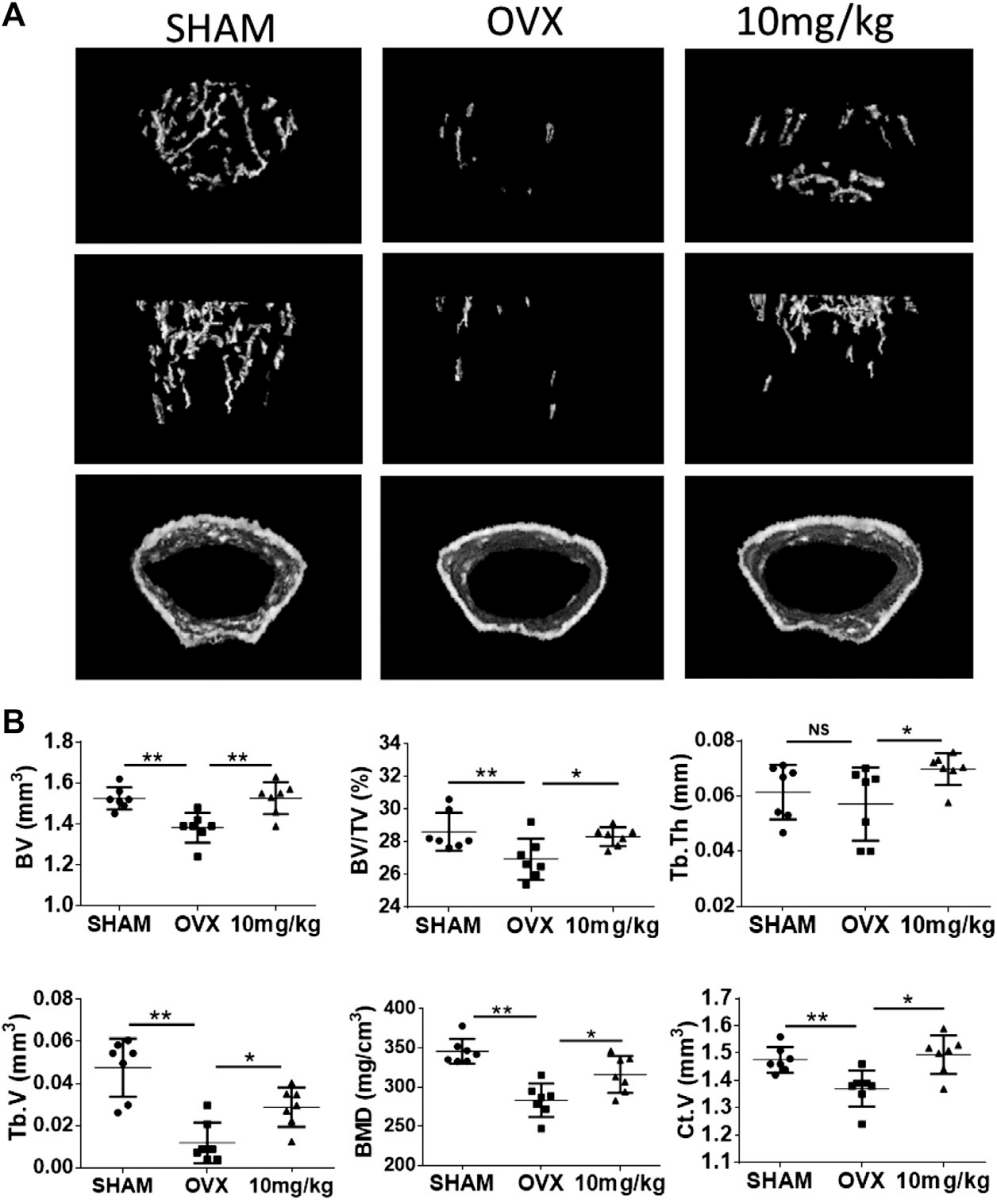
2-NPPA-mitigated bone erosion in a mouse model of OVX-induced bone loss. **(A)** Representative micro-CT reconstruction images of mice in the Sham (*n* = 10), OVX (*n* = 10), and 2-NPPA treatment (*n* = 10) groups. **(B)** Micro-CT analysis of the regions of interest in the tibias and femurs. *p < 0.05, **p < 0.01, and ***p < 0.001 vs. the control group (OVX).

To further investigate the potential preventive effects of 2-NPPA against osteoclast-related bone-resorbing diseases *in vivo*, an OVX mouse model was established (Supplementary Figures S3A,B). Mice body weights of each group were recorded every week (Supplementary Figure S3D). After the isolated bones were fixed, we scanned the whole left femurs and tibias of each mouse by using micro-CT. From the three-dimensional images of the interested regions of the left femurs and tibias, we found that the bone mass in the 2-NPPA-treated group was not lower than that in the control (OVX group), at the same place, as shown in Figure 6A. BV, BV/TV, Tb.Th, Tb.V, BMD, and Ct.V values in the 2-NPPA treatment group were higher (Figure 6B) than those in the OVX group. In addition, the femurs were sectioned and subjected to histological analysis using TRAP staining. Results indicated that the amount of TRAP-positive cells was increased after OVX but decreased after treatment with 2-NPPA (Supplementary Figure S6).

In the serum analysis, result in CTX-1 analysis assay (Supplementary Figure S3C) showed that OVX significantly increased the serum levels of CTX-1; however, 2-NPPA treatment revised the serum CTX-1 level.

## Discussion

Negative regulation of the formation of mature osteoclasts is a vital event for the modulation of healthy skeletal remodeling and the prevention of bone diseases (Jeong et al., 2018). Therefore, it is a critical to examine the signaling pathways at the initiation and termination of the formation of mature osteoclasts from osteoclast precursors (Jeong et al., 2018). In our previous studies, screening of our in-house compound library for anti-osteoclastogenic activity indicated that PPOA compounds could be effective agents for suppressing the formation of mature osteoclasts (Chen et al., 2019b). Subsequently, after screening and comparing the anti-osteoclast formation activity of the effective compounds, our results found that 2-NPPA, a PPOA derivative, exhibited the strongest inhibitory effect on the formation of mature osteoclasts and functioned at the early stage of osteoclast differentiation (Figure 2).

The temporal regulation of RANKL signaling can be classified into several phases, namely initiation, commitment, and termination, based on the signaling process of osteoclast formation (Jeong et al., 2018). In the initiation phase, after stimulation with RANKL and M-CSF, the NF-κB pathway is activated rapidly (within 1 h) through the activation of TRAF6 (Galibert et al., 1998; Wong et al., 1998; Kobayashi et al., 2001). Next, NFATc1 starts to accumulate approximately 1–2 days after RANKL stimulation during the commitment phase. During the final termination phase, RANK signaling regulates cell fusion, which forms the F-actin rings and bone resorption, mainly by NFATc1, which orchestrates the transcription of *Acp5*, *NFATc1*, *DC-STAMP*, *ATP6v0d2*, *MMP9*, and *CtsK*, which are osteoclast-specific genes, together with NF-κB, AP-1, and PU.1 (Boyle et al., 2003; Asagiri and Takayanagi, 2007; Kim and Kim, 2014; Amarasekara et al., 2018). These are all similar to our results (Figures 2, 3). Considering that the effect of 2-NPPA on the formation of osteoclasts occurs at an early stage and evidence suggests that NF-κB, MAPKs and AKT singling pathways played important roles in osteoclast formation (Lee et al., 2016; Wu et al., 2018; Wang et al., 2019) and they are mainly involved in the events that occur in the initiation stage of osteoclast differentiation (Li et al., 2011; Wu et al., 2012), we investigated the activation of NF-κB, MAPKs and AKT signaling within 1 h after treatment. Interestingly, the phosphorylation of NF-κB and I-κBα were dramatically induced after RANKL stimulation, but significantly decreased by treatment with 2-NPPA, as shown in Figure 4A. However, the activation of MAPKs and AKT after RANKL stimulation was not significantly affected by treatment with 2-NPPA (Supplementary Figure S4). The relevant data indicated that the inhibitory effect of 2-NPPA on RANKL-induced bone resorption and F-actin ring formation was partly due to the reduced phosphorylation of NF-κB and I-κBα, which supported the failure of osteoclast formation.

Given the evidence that TRAF6 works upstream of RANKL-mediated signaling (Amarasekara et al., 2018) and, in particular, that the activation of NF-κB signaling in osteoclast differentiation was regulated mainly by TRAF6 (Adhikari et al., 2007; Lamothe et al., 2007a) after the interaction of RANKL with RANK (Amarasekara et al., 2018), we examined the mRNA and protein expression of TRAF6. However, neither the mRNA nor protein expression of TRAF6 was altered after 2-NPPA treatment when compared with the RANKL-stimulated group (Figure 4B, Supplementary Figure S5A). This indicated that 2-NPPA functioned through a different mechanism to other PPOA derivatives. Besides, in the terms of osteoclast-specific gene expressions, compared with other PPOA derivatives induced greatly inhibition in the gene expression, 2-NPPA just showed a minor suppressive in the expression of osteoclast related genes (Chen et al., 2019b). Even for treatment of 2-NPPA, the degree of inhibition of osteoclast-related genes is lower than the degree of inhibition of protein expression (Figures 3–5). All of those implied that 2-NPPA may act post-transcriptionally or post-translationally. A previous study suggested that TRAF6 worked as an E3 ligase in osteoclastogenesis, which activates NF-κB signaling by ubiquitination (Adhikari et al., 2007; Lamothe et al., 2007a). The activation of NF-κB signaling was mediated by lys63-linked TRAF6 ubiquitination (Adhikari et al., 2007; Lamothe et al., 2007a). Our results suggested that 2-NPPA resulted in a slightly reduction in the level of TRAF6-mediated lys63-linked ubiquitination after 2-NPPA treatment (Supplementary Figures S5B–D). Given that 2-NPPA great reduced the formation of osteoclast in 1 μΜ treatment, we can predicted that the inhibitory effect of 2-NPPA on osteoclastogenesis is only partly by reducing TRAF6 or it-mediated lys63-linked ubiquitination.

C-fos, an essential regulator in the formation of osteoclast, is downstream of MAPKs and osteoclasts cannot form in the absence of c-fos (Wang et al., 1992). NFATc1 is a key transcription factor in osteoclastogenesis and report had stated that osteoclast-specific conditional NFATc1-deficient mice developed osteopetrosis due to the defective of osteoclastogenesis (Winslow et al., 2006; Aliprantis et al., 2008). In Figure 4B, the expression of NFATc1 was significant reduced in the 2-NPPA treatment groups during Day1 and Day2, however, a higher level of NFATc1 was appeared at Day3. One possible explanation is that 2-NPPA delayed the formation of osteoclast, subsequently, delayed the degradation of protein NFATc1, since previous paper suggested that the protein of NFATc1 would be degraded during late stage of osteoclastogenesis (Kim et al., 2010). For the c-fos case, our data in Figures 4B,5A showed that the protein expression levels of c-fos were greatly reduced without the interference of its mRNA expression after treating 2-NPPA. Together with the results that overexpression of c-fos diluted the osteoclast differentiation in the treatment of 2-NPPA (Figures 5C–E), indicating that the inhibitory effect of 2-NPPA on mature osteoclast formation is due to the defective of c-fos. Besides, MG132 abolished the inhibitory effect of 2-NPPA on c-fos protein level (Figure 5F). Given the defect in the c-fos protein level in 2-NPPA (1 μM) treatment in Figure 4B and the dramatic inhibition of osteoclast formation via 2-NPPA (1 μM), we can predict that 2-NPPA suppressed osteoclast differentiation by reduction of the protein level of c-fos through proteasomal degradation, which mediated the delayed expression of NFATc1 and subsequently reduced the formation of osteoclast.

Bone homeostasis is mediated by the two vital events of osteoclastic bone resorption and osteoblastic bone formation (Tanaka et al., 2005; Khosla, 2009; Feng and McDonald, 2011; Chen et al., 2017). In the present study, 2-NPPA exerted a small influence on osteoblast differentiation at a treatment concentration of 1 μΜ, but a large inhibitory effect on osteoclast formation, which was confirmed by AR staining after 14 days using primary osteoblasts induced by BMP2 (Supplementary Figures S2A,B). In addition, there was no significant effect on the mRNA expression of Osx and Runx2, the key transcription factors of osteoblast differentiation (Rahman et al., 2015), after 2-NPPA treatment (Supplementary Figure S2C,D). Based on the current data (Supplementary Figure S2) it can be predicted that 2-NPPA have a little effect, even does not effect, on bone formation *in vitro* and *in vivo*. Finally, an OVX-induced bone loss model was set up to evaluate the *in vivo* effect of 2-NPPA on bone erosion. As expected, 2-NPPA (10 mg/kg) treatment significantly reduced the bone loss induced by surgery compared with the OVX group (solved-treated), as shown by increases in BV, BV/TV, Tb.Th, Tb.V, Ct.V, and BMD measured by micro-CT. All this evidence demonstrated the therapeutic potential of 2-NPPA for the treatment of bone loss-related disorders. However, further investigation still are need to evaluate and confirm the effect of 2-NPPA on bone formation *in vivo* in the future.

In summary, the findings of our study are that 2-NPPA attenuated RANKL-induced osteoclastogenesis via reduction of the phosphorylation of NF-κB, I-κBα and the defective of c-fos through proteasomal degradation, which mediated the delayed expression of NFATc1 and subsequently reduced the formation of osteoclast and ameliorated bone loss. Hence, our results presented proof-of-concept for the use of 2-NPPA as a novel agent for anti-resorptive bone loss in mice.

## Data Availability Statement

The raw data supporting the conclusions of this article will be made available by the authors, without undue reservation, to any qualified researcher.

## Ethics Statement

The animal study was reviewed and approved by All animal experiments were approved by IACUC at Chonnam National University (approval number: CNU IACUC-YB-2019-46).

## Author Contributions

Conceptualization: T-HL and SL; methodology: ZC, EC, JS, MD, SL, and T-HL; investigation: ZC and MD formal analysis: MD and ZC; writing: ZC, EC, and T-HL.

## Funding

This research was supported by the Bio and Medical Technology Development Program of the National Research Foundation (NRF) funded by the Ministry of Science, ICT and Future Planning (NRF-2016M3A9B6903087), and by the Korea Mouse Phenotyping Project of the Ministry of Science, ICT and Future Planning through the National Research Foundation (NRF-2014M3A9D5A01073658, KMPC). The funders had no role in the design of the study; in the collection, analyses, or interpretation of data; in the writing of the manuscript, or in the decision to publish the results.

## Conflict of Interest

The authors declare that the research was conducted in the absence of any commercial or financial relationships that could be construed as a potential conflict of interest.
