# Supplementary material for: 2-NPPA Mitigates Osteoclastogenesis via Reducing TRAF6-Mediated c-fos Expression
Source: Front Pharmacol. 2021 Jan 26;11:599081. doi: 10.3389/fphar.2020.599081 (PMC7870508; doi:10.3389/fphar.2020.599081)
Supplement: Supplementary file 1 [file table1.doc]

**SUPPLEMENTARY INFORMATION**

**Supplementary Table S1. Primers used in this study**

| Gene(mouse) | Primer sequence (5' to 3') |  |
| --- | --- | --- |
| GAPDH | Forward | TGTGTCCGTCGTGGATCTGA |
|  | Reverse | GATGCCTGATTCACCACCTT |
| Cathepsin K | Forward | ACTTCCGCAATCCTTACCGA |
|  | Reverse | TTCGCTAGGCTCTTTTCGGA |
| DCSTAMP | Forward | CGCACGATGCTTCATTCTTC |
|  | Reverse | CAGTGCCAGCCGCAATC |
| ACP5 | Forward | TTTATGCTGGACACAGTGATGCT |
|  | Reverse | CCCAGGTCTCGAGGCATT |
| NFATc1 | Forward | ACCACCTTTCCGCAACCA |
|  | Reverse | GGTACTGGCTTCTCTTCCGTTTC |
| Atp6v0d2 | Forward | GTGAGACCTTGGAAGACCTGAAA |
|  | Reverse | TCCTCATCTCCGTCAATTTTG |
| MMP9 | Forward | CTGGACAGCCAGACACTAAAG |
|  | Reverse | CTCGCGGCAAGTCTTCAGAG |
| TRAF6 | Forward | TCGGACCCTGGAGGACAA |
|  | Reverse | CCAAACTTGCCAATCTTCCAA |
| Osterix | Forward | GGTCCCCAGTCGAGGAT |
|  | Reverse | CTAGAGCCGCCAAATTTGCT |
| Runx2 | Forward | ACTATGGCGTCAAACAGCCT |
|  | Reverse | GGTGCTCGGATCCCAAAAGA |


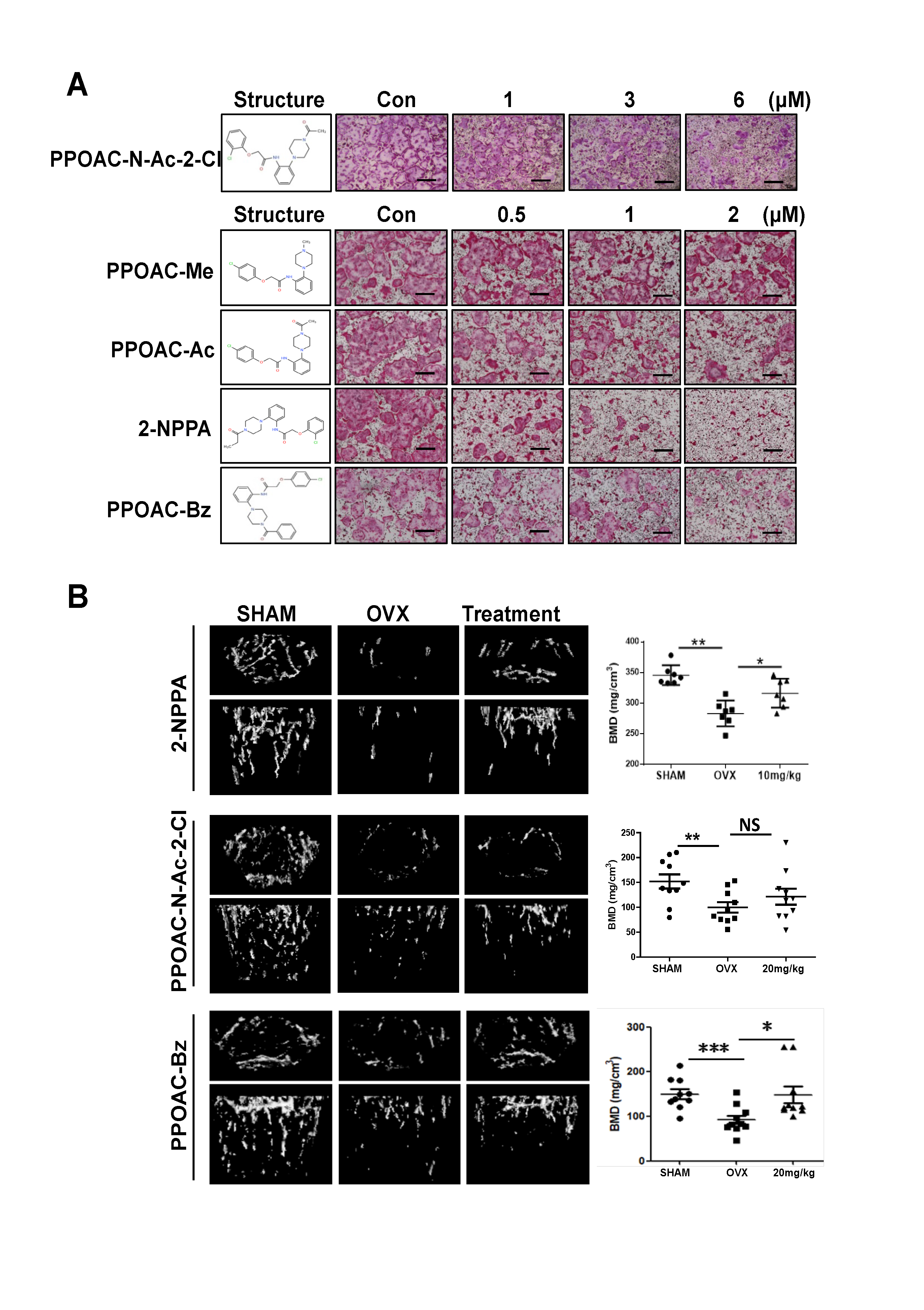


**FIGURE S1│** 2-NPPA showed a stronger inhibitory effect on RANKL-induced osteoclastogenesis compared with the indicated derivatives *in vitro* and *in vivo*. (**A**) Structure of PPOA-*N*-Ac-2-Cl and the inhibitory effect of PPOA-*N*-Ac-2-Cl (0, 1, 3, and 6 μM) on osteoclast differentiation, as shown by TRAP staining. The structures of 2-NPPA and its derivatives; the inhibitory effect of 2-NPPA and its derivatives (0, 0.5, 1, and 2 μM) on osteoclast differentiation, as shown by TRAP staining. BMMs were treated with the indicated doses of PPOA-*N*-Ac-2-Cl, 2-NPPA, and its derivatives in induction medium containing 30 ng/mL M-CSF and 50 ng/mL RANKL; After the mature osteoclasts were formed in the control (Con) group, TRAP staining was performed to visualize the formation of osteoclasts. (**B**) Representative micro-CT reconstruction images and analyses of the regions of interest in the tibias and femurs of mice in the SHAM, OVX, and treatment groups of the indicated compounds. * p <0.05, ** p <0.01, and *** p < 0.001 vs. the control group, OVX. (The pictures of the published compounds used in **FIGURE S1** are same with our previously paper: “https://www.mdpi.com/1422-0067/20/20/5196”, micro-CT images of 2-NPPA is same with **FIGURE 5**)


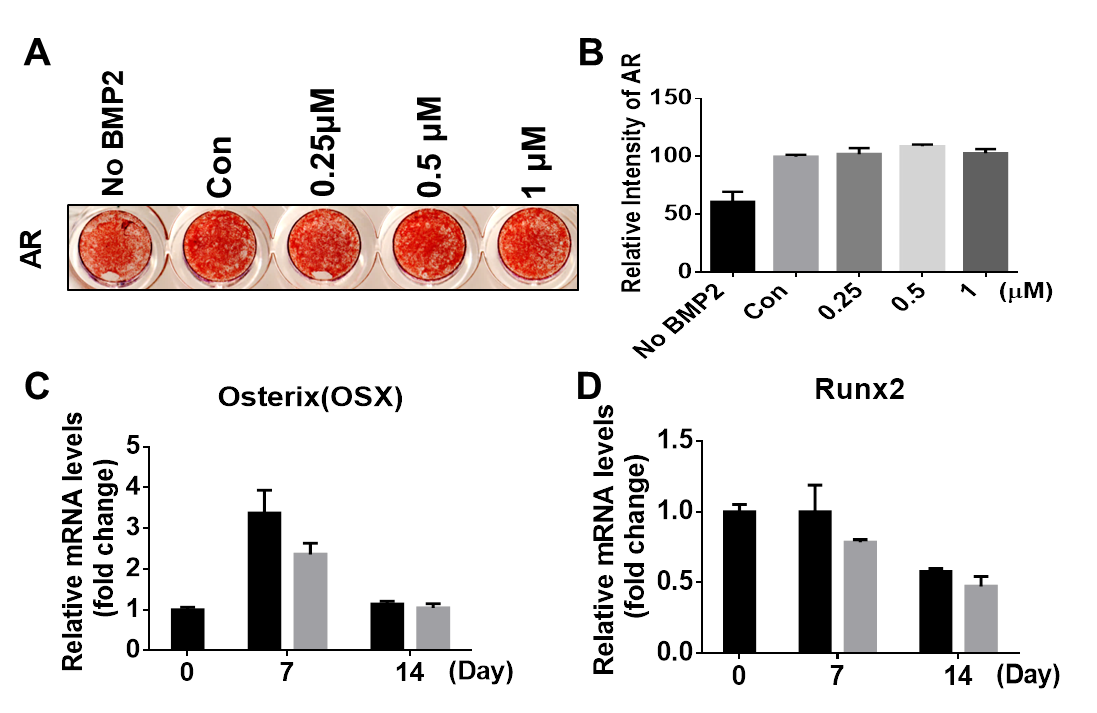


**FIGURE s2│** 2-NPPA has a little effect on BMP2-induced osteoblast differentiation in vitro. (**A**) Primary osteoblast was isolated from the calvaria of 3 days mice pubs, after induced for osteoblast differentiation with or without using 100ng/ml BMP2, supplied with the indicated doses of 2-NPPA (**A**) the AR staining was performed on the day14. The intensity of AR staining (**B**) of the indicated groups were calculated using ImageJ. “Con” indicates only BMP2 treatment. Real-time PCR was perform to examine the mRNA levels of the Osx (**C**) and Runx2 (**D**) on the day7 and day14. Transcript levels were normalized to the expression of the control at Day 0. The data are presented as the mean ± SD of three independent experiments.


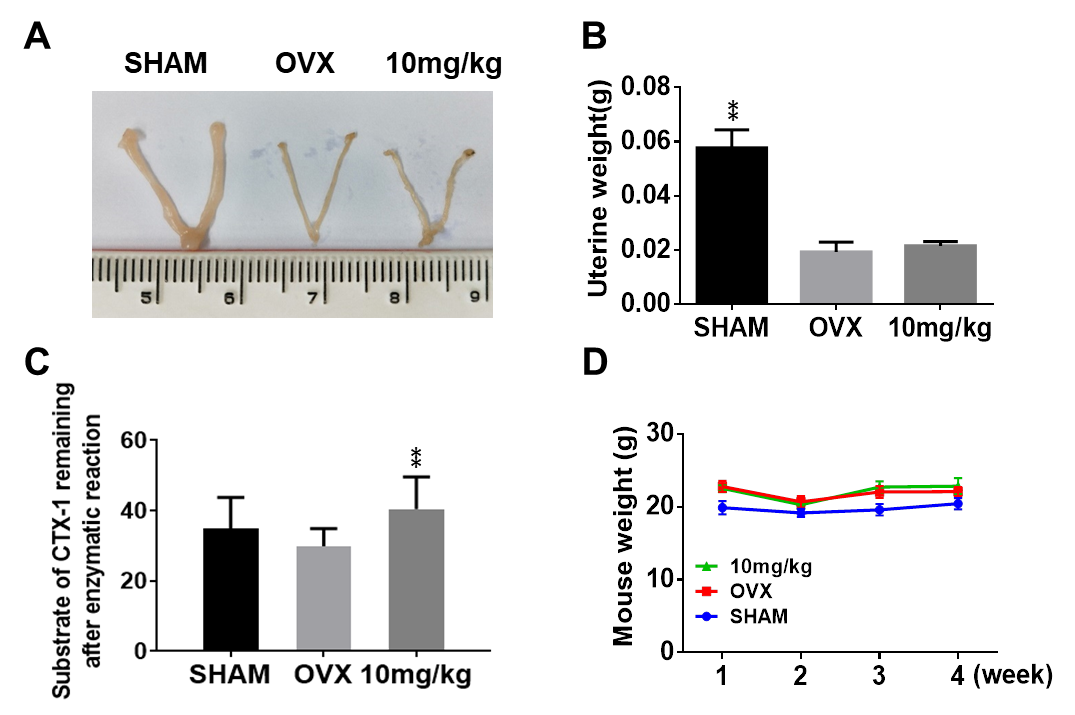


**FIGURE s3│** successful of the OVX-induced bone loss model. (**A**) Uterine of the sham, ovx and 2-NPPA groups. (**B**) Uterine weight of the sham, ovx and 2-NPPA groups. (**C**) CTX-1 enzymatic reaction assay. (**D**) Mice body weight of each group for *in vivo* experiment. ** *p* < 0.05 versus the vehicle-treated control , OVX. Each group, n=10.


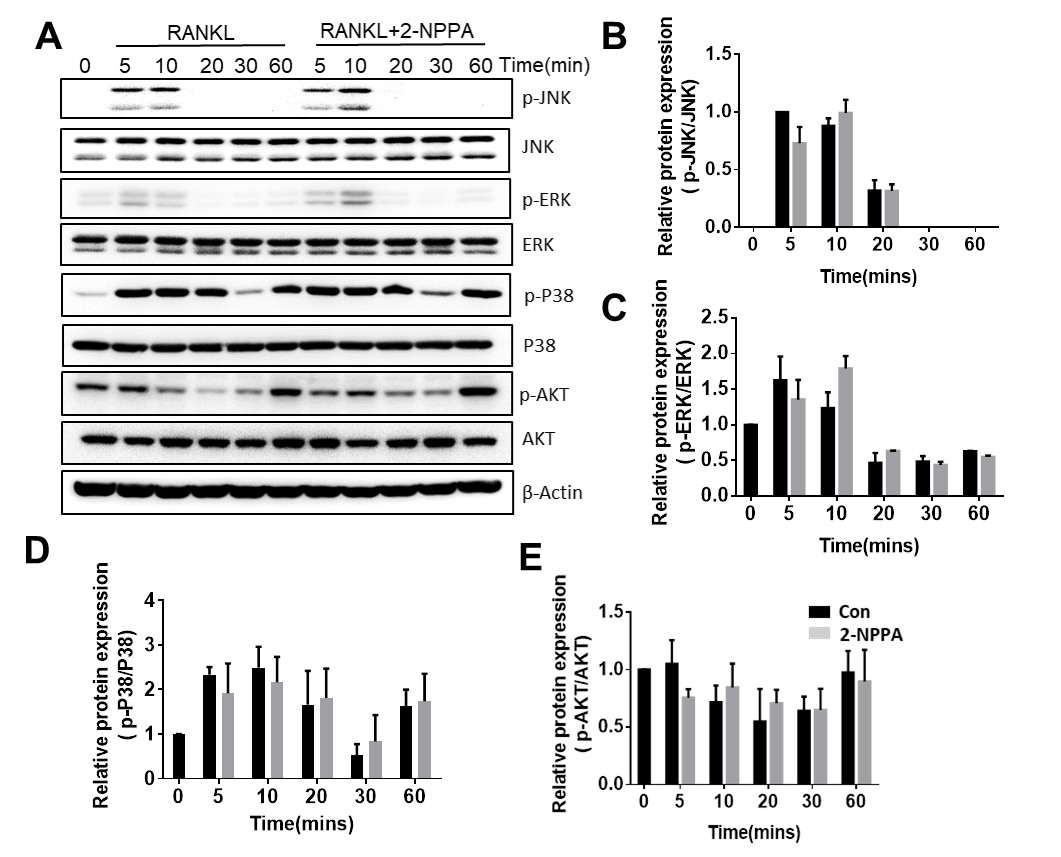


**FIGURE s4**│ 2-NPPA has little influence on the activation of MAPK and AKT signaling pathways. (**A**) BMMs were treated with 100 ng/mL RANKL for 0, 5, 10, 20, 30, or 60 min together with 1 μM 2-NPPA or DMSO, and the phosphorylation of JNK, ERK, P38, and AKT were analyzed by using immunoblotting. (**B-E**) The densitometry graphs of (A); β-actin was used as a loading control. The data are presented as the mean ± SD of three independent experiments.


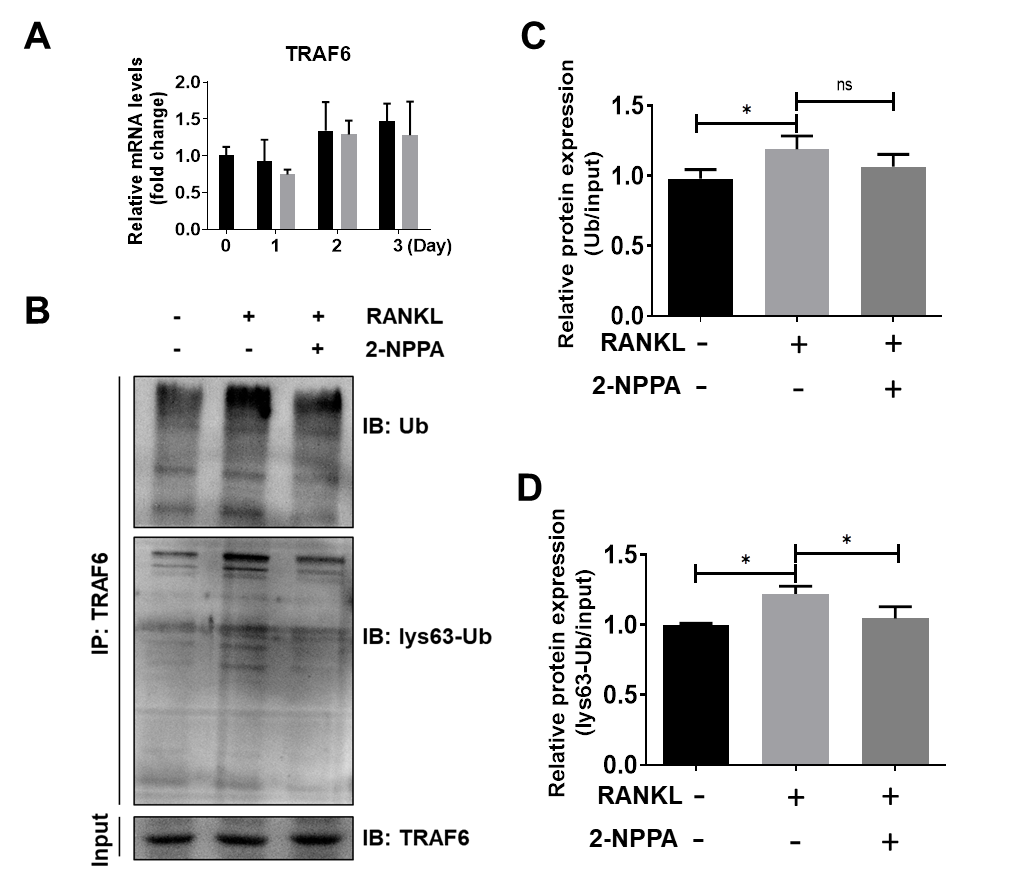


**FIGURE s5│** 2-NPPA slightly effect on the TRAF6-mediated lys63 linked-ubiquitination. **(A)** Real-time PCR was perform to examine the mRNA level of the TRAF6. (**B**) BMMs incubated in the indicated conditions and immunoprecipitation carried out with TRAF6 antibody. Immunoprecipitates subjected to western blot analysis with the indicated antibodies. (**C-D**) The densitometry graphs of (B); Inputs were used as loading control. * *p* <0.05, NS indicated “no significant”. The data are presented as the mean ± SD of three independent experiments.

**
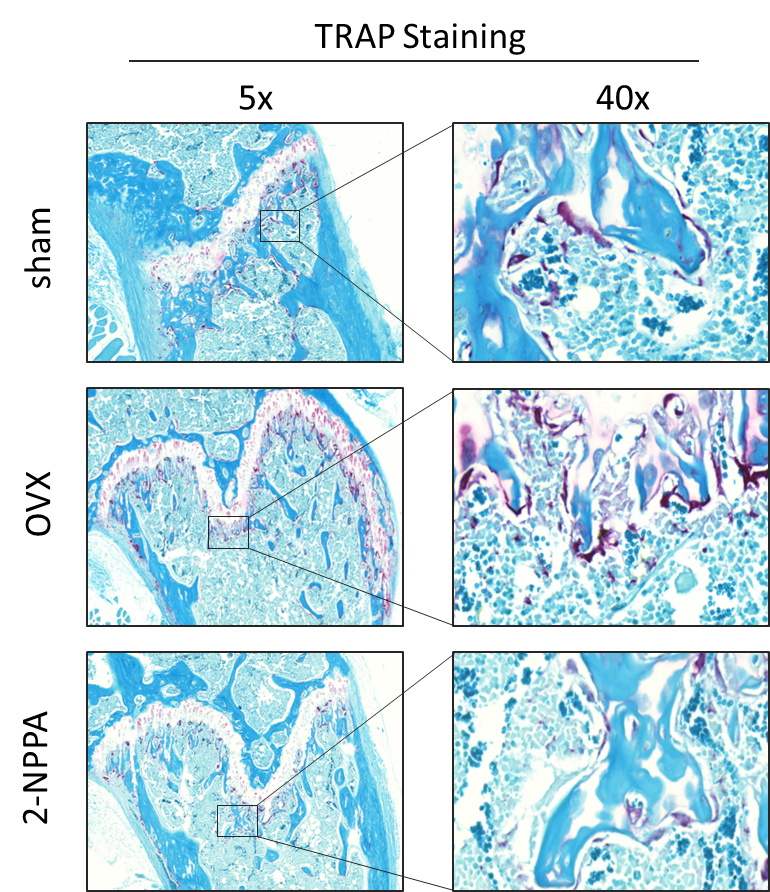
**

**FIGURE s6**│ Analysis of osteoclast activity in vivo in the presence or absence of 2-NPPA. Mouse (n=10) femur sections were fixed, decalcified, and sectioned. TRAP staining was used to indicate the TRAP-positive cells around the bone (fast green staining was applied to provide a background).
